# Smartphones and Varsity Athletes: A Complicated Relationship

**DOI:** 10.3389/fspor.2020.560031

**Published:** 2021-01-06

**Authors:** Poppy DesClouds, Natalie Durand-Bush

**Affiliations:** SEWP Lab, School of Human Kinetics, Faculty of Health Sciences, University of Ottawa, Ottawa, ON, Canada

**Keywords:** athlete, varsity, sport, smartphone, technology, social media, focus groups

## Abstract

Varsity athletes are a group of high performers situated within a demographic notable for smartphone usage and media-multitasking. Surprisingly, little research has examined the impact of smartphones in the lives of varsity athletes. The purpose of this exploratory, qualitative study was to begin addressing this gap by investigating varsity athletes' experiences with smartphones. Varsity athletes (*n* = 21) from nine different sports participated in one of five focus groups, and data emerging from these discussions were subjected to an inductive thematic analysis. Results indicate that smartphones are a mainstay of varsity athletes' experiences, as the athletes regularly use their smartphones to manage roles and demands across multiple contexts (e.g., sport, school, home). Themes pertained to concurrent negative (e.g., stress, distraction, disengagement) and positive (e.g., self-regulation, social connectedness) implications of smartphone usage, making it clear that athletes' relationship with their smartphone is a complicated one. Findings contribute to the limited studies of smartphone usage among athletes, and support the notion that implications of usage exist along a continuum, rather than in distinct categories of “good” and “bad”. Results can inform practical guidelines for optimising athletes' use of smartphones in and around the sport context.

## Introduction

Smartphones are omnipresent in modern society, particularly among young users. In Canada, 98% of 15–24-year-olds own a smartphone (Statistics Canada, 2019), and the number of smartphone users has continued to climb despite slowing trends in other areas of the world (Deloitte, 2019). There is no doubt that smartphones can be both “good” and “bad,” as they allow us to stay connected to the world, but can also lead us to feel distracted and frustrated (Smith, 2015). Not surprisingly, there is a great deal of health, psychology, communications, and education research related to the impact of smartphone usage. However, there is a deficit of literature in the sport psychology domain pertaining to the ways in which smartphones are changing and impacting the experiences of athletes (Durand-Bush and DesClouds, 2018). Athletes, like the rest of the population, face the task of negotiating their smartphone usage on a day-to-day basis. This is particularly true for varsity athletes, a young subset of high-performance athletes, who have grown up training, living, and learning with mobile devices at their sides. Anecdotal evidence shows that varsity athletes are using smartphones (e.g., Gregory, 2019), but the question remains: How are varsity athletes using their smartphones, and does their usage positively or negatively impact their experience of being a varsity athlete?

## Smartphone Usage

The literature surrounding smartphone usage is rapidly evolving, as are the features and functions of smartphone technology. Reports on various cohorts show smartphone usage averaging over 2.5 h per day (e.g., Smith, 2015; Deng et al., 2019) accumulated through extremely short and sporadic periods of usage (Bentley et al., 2015; Deng et al., 2019). Young users are particularly smartphone savvy and deeply intrenched in usage (Bentley et al., 2015; Smith, 2015; Twenge, 2017). The “iGeneration” (iGen; those born in the 1990s onward) comprises individuals who have been privy to mobile access to the internet since adolescence (Rosen, 2011; Twenge, 2017). Members of this cohort are notable for media consumption, social media usage, electronic communication and socialisation, media multitasking, fear of missing out (FOMO), and digital distraction (Rosen, 2011; Przybylski et al., 2013; Rosen et al., 2013; Twenge, 2017). Prone to separation anxity when they are without their devices (Cheever et al., 2014), many in this demographic turn to their phones in any spare second, especially when bored and in waiting times (Bentley et al., 2015; Smith, 2015).

### Impact of Smartphone Usage

In a breadth of disciplines, research related to the impact of smartphones is ongoing. The literature surrounding the impact of smartphones is largely unsystematic and a uniform definition of smartphone usage is not employed. Moreover, many studies pertain generally to screen time, with the smartphone as only one component of media usage, or they pertain only to a specific feature of usage (e.g., social media, mobile health interventions), without clarifying whether a smartphone was used to access this feature. In order to gain a broad understanding of the positive and negative psychosocial implications of smartphone usage, while also acknowledging the prevalent use of smartphones to access social media (Smith, 2015), this review of literature includes research related not only to smartphone usage, but also specific features such as health intervention and social media applications.

Research from other domains shows us that smartphone usage can be both positive and negative, and accumulating evidence is beginning to suggest an interplay of negative and positive effects. Table 1 summarises these effects.

**Table 1:** Positive and negative implications of smartphone usage.

**Positive implications of usage**	**Negative implications of usage**
**Increased/Enhanced**	**Decreased/Hindered**
• Social connectedness (Best et al., 2014; Gardner and Davis, 2014; Ryan et al., 2017) • Communication (Gardner and Davis, 2014; Bentley et al., 2015) • Identity and image management (Gardner and Davis, 2014; Chan et al., 2015; Park et al., 2020) • Self-disclosure (Best et al., 2014; Gardner and Davis, 2014) • Social and emotional support (Wright, 2012; Best et al., 2014; Frison and Eggermont, 2015) • Well-being (Wright, 2012; Best et al., 2014) • Learning (e.g., Chan et al., 2015; George and DeCristofaro, 2016) • Self-regulatory behaviours (e.g., Quelly et al., 2015)	• Cognitive capacity, attention, analytic thinking, and working memory (Abramson et al., 2009; Stothart et al., 2015; Ward et al., 2017; Marty-Dugas et al., 2018) • Sleep (Thomée et al., 2010) • Task performance and information processing (Stothart et al., 2015; Carrier et al., 2018; Fortes et al., 2019) • Productivity and efficiency (Rosen et al., 2013; Carrier et al., 2018) • Social presence (Chotpitayasunondh and Douglas, 2016; McDaniel and Coyne, 2016) • Self-control (Abramson et al., 2009; Rosen et al., 2013; Carrier et al., 2018) • Well-being (Best et al., 2014; Rosen et al., 2014)
	**Increased/Exacerbated**
	• Stress/anxiety (Thomée et al., 2010; Clayton et al., 2015; Elhai et al., 2017; Vahedi and Saiphoo, 2017) • Mental health concerns (Elhai et al., 2017, 2019; Vahedi and Saiphoo, 2017) • Mental fatigue and cognitive load (Thomée et al., 2010; Greco et al., 2017; Fortes et al., 2019) • Isolation and loneliness (Thomée et al., 2010; Best et al., 2014; Gardner and Davis, 2014; Ryan et al., 2017)

An important distinction can be made between debilitative and facilitative (i.e., normal) smartphone usage, based on the type, purpose, and intent of usage (Marty-Dugas et al., 2018; Elhai et al., 2019; Ellis, 2019). Facilitative usage tends to be productive, purposeful, and intentional, and it does not impede regular functioning or cause distress (Elhai et al., 2019). An important focus, then, beyond the amount of usage, is the intent and impact of smartphone usage in one's daily life. Emerging reviews of smartphone research recommend the acceptance of smartphones and consideration of the broader purpose, type, and context of usage when researching smartphone technology (e.g., Ellis, 2019).

### Smartphones and Sport

There are only a handful of studies in which athletes' use of smartphones and accompanying features (e.g., social media, training apps) have been examined. Some have started to address the potential implications of smartphone usage on athletes' performance. These studies suggest that athletes' use of smartphones and/or social media at various stages prior to sport performance can disrupt concentration (Encel et al., 2017), inhibit decision-making (Fortes et al., 2019), induce mental fatigue (Greco et al., 2017; Fortes et al., 2019), and delay sleep (Jones et al., 2019), in some instances, leading to performance decrements (Greco et al., 2017; Fortes et al., 2019; Jones et al., 2019). A distinct, but related stream of research has focused on competitive athletes' self-presentation and sharing behaviours on social media (e.g., Smith and Sanderson, 2015; Nankervis et al., 2018), as well as experiences and implications of using various platforms, particularly Twitter (David et al., 2018; Sanderson, 2018; Park et al., 2020). Ever-evolving social media usage continues to introduce new complexities to the varsity sport context, as these platforms provide athletes with great autonomy for identity management, communication, and public sharing. However, they equally pose risk to athletes' performances and experiences, and the curated public image of athletes and post-secondary sport programs. Taken together, these studies pertaining to the social media usage of athletes point to both positive (e.g., team support, motivation, image management, and connexion), and negative (e.g., criticism, obligation, and anxiety) experiences and implications. Importantly, these studies focus explicitly on social media, which is only one aspect smartphone usage. Thus, while this stream of research provides valuable insights, it overlooks the technology used to access social media, and accounts for only a small portion of the possible smartphone usage behaviours and implications.

Athletes' lived experiences with smartphones, and the positive and negative impact of using these devices in and around the sport context remains unclear. This is concerning, considering that many high-performance athletes are part of the iGen, known for outstanding and nuanced smartphone usage. A preliminary study by DesClouds et al. (2018) showed that a group of Canadian varsity athletes were using their phones for an average of 32 h per week. This is more than one full day per week dedicated to smartphone usage alone. The athletes' most used applications pertained to social media. In fact, the weekly usage time for social media exceeded the use of any other application by 7 h on average. This study suggests that varsity athletes are using smartphones a great deal in their day-to-day lives. More evidence is required, as the impact of smartphone usage on athletes has rarely been empirically investigated. In an attempt to address this gap, the purpose of this exploratory study was to investigate varsity athletes' lived experiences with smartphones. More specifically, the aim was to gain an in-depth understanding of varsity athletes' positive and negative smartphone experiences by investigating the core research question “How are varsity athletes using their smartphones, and how is their usage impacting their experience of being a varsity athlete?”

## Materials and Methods

There is a paucity of research related to the smartphone usage behaviours of varsity athletes, as well as the implications of smartphone usage in these athletes' lives. Therefore, an exploratory, qualitative study was employed to uncover the pertinent elements of smartphone usage within Canadian varsity athlete cohorts. This study was guided by the critical realist paradigm. A critical realist approach promotes the use of dialogic methods, and assumes that an unknowable reality is partially accessible only through the perceptions and interpretations of individuals (DeForge and Shaw, 2012; Fletcher, 2017). It also recognises the importance of participants' interpretations of the subject being studied, and leaves it to the researcher to use existing theory and empirical work to negotiate and eventually consolidate multiple perspectives into the best possible understanding of reality (DeForge and Shaw, 2012; Fletcher, 2017).

### Focus Groups

In line with a critical realist approach, the data for this study were collected through focus groups. Focus groups encouraged dialogue and discussion among participants, and allowed the researchers to access the participants' interpretations of the phenomenon under study (i.e., smartphones) in a specific context (i.e., varsity athletes). The focus groups helped the researchers to gain a broad understanding of varsity athletes' experiences, while drawing attention to specific examples and nuances within their shared world. Both breadth and depth of data were deemed important in this exploratory study to lay a foundation for inquiry, as well as to inform future studies. Focus groups are often used as a preliminary step in exploratory research programs, and can also stand alone as a research method in order to explore a novel or unfamiliar research topic (Wilkinson, 1998). Given the novelty of the investigation of smartphones in the context of varsity sport, focus groups were deemed to be the most acceptable method to allow for the identification of both individual and collective perspectives and experiences, in an interactive and efficient manner. In line with a critical realist approach, the typologies developed from the data analysis were based on negotiation and consolidation of the language of the participants, the researchers' interpretation of the data, as well as the existing literature (Fletcher, 2017).

### Context

The varsity sport context is arguably an ideal one in which to examine the impact of smartphone usage. Varsity athletes represent not only a demographic deeply situated within the iGen, but also a unique group of athletes required to perform and self-regulate at a high-level in both sport and school (Dubuc-Charbonneau and Durand-Bush, 2015). For example, Canadian varsity athletes are required to maintain a high academic standing alongside strong sport performance outcomes, particularly if they are seeking or holding scholarships. This can create added demands and pressure that could impact athletes' behaviours. Indeed, the literature points to both benefits and drawbacks of participating in varsity sport. Positive outcomes include the development of self-esteem, discipline, social networks, teamwork, and leadership (Watt and Moore, 2001). On the other hand, drawbacks include the management of complex, conflicting roles and schedules, feelings of isolation and frustration, and pressure to maintain performance standards (Watt and Moore, 2001; Gould and Whitley, 2009).

It is no surprise, then, that the demands put on student-athletes can induce stress, burnout, and illness (Gould and Whitley, 2009; Dubuc-Charbonneau and Durand-Bush, 2015). The potential for distractions, setbacks, and compromised well-being is high if athletes cannot effectively self-regulate (Dubuc-Charbonneau and Durand-Bush, 2015). Given the benefits and drawbacks of smartphone usage that have been identified in other domains, one could hypothesise that the use of smartphones in the varsity sport context could both facilitate and hinder athletes' capacity to self-manage multiple roles and demands, maintain well-being, and achieve goals across school, sport, and social contexts. For all these reasons, varsity sport was deemed an ideal context in which to carry out the proposed project.

### Participants

Participants were recruited from two universities in Canada, each with an established varsity sport program including various individual and team sports. Using a purposive sampling technique (Patton, 2002), participants were recruited through social media, as well as through informational emails sent to university sport services departments, which were then distributed to athletes at the discretion of sport service directors and coaches. Snowball sampling also occurred, through word-of-mouth from other focus group participants. Participants had to be current student-athletes competing in a Canadian varsity-sanctioned sport. Ethics approval was granted from both universities involved. Consent of the athletic directors at each institution was obtained prior to recruitment activities, and all study participants signed a consent form and were provided with a copy for their own records. A total of 21 athletes (9 men, 12 women) from nine different sports (i.e., track and field, basketball, rowing, fencing, Nordic ski, rugby, swimming, lacrosse, and soccer) took part in the study. Their year of varsity eligibility ranged from first to fifth year. It is noteworthy that at the time of the study, both institutions' sport departments had policies accounting for the social media usage of their varsity athletes, but not the use of smartphones. These policies pertained to respecting and representing the ethics, values, and integrity of the institution. Only one policy made clear that athletes would be subject to sanctions if they did not abide by regulations. These policies were presented only once to participants during their varsity sport orientation at the beginning of the school year.

### Procedure

Five focus groups each comprising three to five participants (*M* = 4.2) were conducted in-person at the host institution, in a private room. The duration of the focus group discussions ranged from 64 to 109 min (*M* = 85.2). The number of participants in each group allowed the exploration of a variety of perceptions and opinions (i.e., breadth), and the identification of both individual and collective perspectives and experiences in an interactive and efficient manner (i.e., depth; Rio-Roberts, 2011). Athletes were recruited for focus groups until the researchers could make a strong case that an acceptable level of saturation had been reached. Saturation was reached in the fifth focus group, insofar that the research question could be appropriately answered, the story regarding athletes' experiences had taken shape, topics of discussion were becoming repetitive and familiar, and further inquiry might have been counter-productive to the research objective (Mason, 2010).

Focus groups were held during the Fall and Winter semesters, so participants would be actively involved in both school and sport at the time of the interviews, as well as in both training and competition periods. The focus groups were guided by an in-depth, semi-structured interview guide, and were led by the lead researcher who is a Mental Performance Consultant and well-versed in interviewing. Steps were taken to reduce effects of social desirability, which included assuring participants that their responses would be anonymized and any information linking to their name, sport, or institution would be removed. It was also emphasised that all responses should remain confidential within the group. Participants were informed that the researchers were inquiring about both negative and positive experiences, so there would be no “right” or “wrong” answers, nor a systematised order of responses. At the beginning of each focus group, athletes were reminded that they could choose to answer or not answer questions at their own discretion, and discussion among participants was encouraged.

### Interview Guide

A multi-section interview guide was used to direct the semi-structured focus groups. The guide was flexible and promoted “responsive interviewing” (Rubin and Rubin, 2012), whereby a continuous give-and-take between the interviewer and respondents was encouraged to maintain direction without overbearing structure. Participants were encouraged to embark on discussion with one another, and to give concrete examples of experiences with their smartphones.

The first and second sections of the guide pertained to introductions and motivations to participate, so members of the focus group could build rapport and become comfortable with one another, and the interviewer (e.g., “When did you start playing your sport?”). The third section pertained to participants' experiences and perceptions of varsity athletics (e.g., “What does it mean to be a varsity athlete?”), and the fourth section dealt with participants' preference and priorities when it came to smartphones (e.g., “Do you consider your smartphone to be an essential tool? Can you live without it? Explain.”). These sections were used to set the stage for an in-depth, context-specific discussion.

The fifth section honed in on participants' smartphone usage, including perceptions of usage, prevalence, and context of usage. This was first established generally (e.g., “Primarily, what do you use your smartphone for?”), and then in-depth with questions related to usage for sport, academics, and communication, respectively (e.g., “What smartphone features do you use most often when you are at training, practise and/or competition? Explain.”). In the sixth section, participants focused in on domains of sport, school, and social life, as they discussed questions such as, “What role does your smartphone have in your life?” and, “What smartphone rules or restrictions do you have to abide by?” The final section provided the athletes an opportunity to reflect on the focus group discussion, share additional experiences, and make final comments.

### Data Analysis

Guided by critical realist assumptions, it was important that participants' perspectives and interpretations were emphasised first, and then compared and consolidated by the researcher with existing empirical data. As such, an inductive, reflexive thematic analysis of the focus group data was performed. This method was chosen in order to thoroughly describe and understand the data, while also emphasising the context of the phenomenon under study. In line with a critical realist approach, reflexive thematic analysis is a method that relies on researchers to engage in continued reflexive practise and re-negotiate their analyses of the data in order to consolidate the best possible interpretation of reality. Researcher subjectivity is deemed to be a “resource” for informing data analysis, and transparency is paramount (Braun and Clarke, 2019). Therefore, based on existing literature in other domains, the lead researcher approached this dataset with the assumption that polarised terms of “negative vs. positive” were appropriate initial organising concepts to navigate the phenomenon of smartphone usage among athletes.

In addition to the inductive, reflexive component, a data-driven coding process was undertaken to stay true to the exploratory goals of the research (Braun et al., 2016). The analysis was guided by Braun and Clarke's (2006, 2019) recommendations. The lead researcher first immersed herself in the data by listening to the focus group audio recordings and by reading through the transcripts for deep familiarisation. She recorded initial ideas, comparisons, and reactions, and noted inter-group dynamics describing emphasis, emotion, or unique qualities. This aided her in understanding group characteristics when discussing helpful/positive and debilitative/negative smartphone usage experiences. Verified transcripts and researcher notes were then imported into NVivo software for organisation, coding, and analysis, which allowed the researcher to perform complex searches and keep an audit trail.

The researcher parsed the raw interview transcripts into initial descriptive codes representing patterns of shared meaning among the athletes. After coding all of the data, she meaningfully grouped the initial codes into high-order themes related to the central organising concepts of positive vs. negative implications. These themes represented the experiences and perceptions of smartphone usage that were common or shared among the athletes, as well as any outlying or contrasting perspectives and experiences. The researcher worked reflexively throughout her development and organisation of themes in order to question and re-negotiate her own definitions and interpretations of the dataset. Cyclically, the researcher reflected on and reworked several iterations of the thematic structure so as to best represent the data as it related to the core research question. To enforce qualitative rigour, the thematic structure was checked by “critical friends” (Smith and McGannon, 2018), through a series of formal meetings with the second author and research peers. The second author (the first author's PhD supervisor) critically explored the coding tree and high-order themes from the perspective of someone with in-depth knowledge of the competitive sport community, but at arm's length from the focus groups and raw research data. This process of critical friends helped with interpretation of the data and consideration of alternative perspectives and feedback. In the end, three high-order themes were constructed by the researcher, with eight sub-themes representing patterns of meaning among the varsity athletes related to positive and negative implications of smartphone usage.

## Results

The results are presented in three sections: general usage, negative usage, and positive usage. All three sections pertain to varsity athletes' perceptions of the positive and negative experiences of smartphone usage in and around the sport context. Participant identification codes are provided with each citation (F = Female, M = Male; T = Track and Field, B = Basketball, Rw = Rowing, X = Nordic Ski, R = Rugby, Sw = Swimming, L = Lacrosse, S = Soccer, F = Fencing), and numbers indicate the focus group in which the athlete participated. For example, a female rugby player from the third focus group was coded as RF-3.

### General Usage

General usage pertains to athletes' usage preferences, behaviours, and experiences that describe the nature and context of smartphone usage in the varsity sport setting.

#### Characteristics of Usage

The athletes most frequently reported using their smartphones to access Facebook, Messenger, Instagram, Snapchat, YouTube, music, and organisational tools such as calendar, alarm, and e-mail applications. There was a divide between Android and iPhone users; out of the 21 athletes, 10 used an Android device, predominantly due to affordability. Participants were asked to classify themselves as heavy, moderate, or light smartphone users and then to provide an explanation of their self-classification. Overall, athletes self-identified as heavy, moderate, or light smartphone users, with the majority of participants referring to themselves as moderate or heavy users (*n* = 17; 81%). Heavy users reported having their smartphone on them or near them at all times, and using their device for “everything” throughout the day. They described the need to check and respond to notifications constantly and with immediacy. Moderate users identified themselves in terms similar to that of heavy smartphone users, with the caveat that they regularly tried to monitor their smartphone usage, and reduce unhelpful smartphone habits. Conversely, light users (*n* = 4; 19%) identified only feeling the need to use their phone for essential tasks, and otherwise, felt able to separate from and ignore their device, feeling no pressure to answer texts, calls, or notifications.

Athletes also discussed using their smartphones to occupy themselves when bored, filling in time, or procrastinating from completing uninteresting tasks: “During competitions, in between events, I'll usually just text people so I have something to do” (TF-1). They attributed much of their usage to the physical habit of picking up their phone, simply because it is always there: “It's in your sports bra. It's [with you] every time you work out. The only place it's not [with you] is in the shower, really. And even then!” (RF-2). The athletes found themselves to be using their smartphones most in the “to and from” and “waiting” times in the sport context (e.g., in transit to and from training or competition, waiting for the next heat or game).

#### Awareness and Nature of Usage

The athletes showed an acute awareness of their own smartphone usage, as well as others' smartphone usage, particularly the negative implications of usage across the various contexts of their lives (i.e., sport, school, and social). The athletes were in-tune with their internal dialogue warning them of their own “bad” media habits and negotiating self-control. Athletes consistently returned to social media during the focus group conversations. They passionately discussed the nature of social media in their sporting lives, and reported using social media for purposes of entertainment, self-promotion, motivation, comparison, and self-presentation, particularly “authentic” and “athletic” self-presentations. One athlete described, “People can put up two faces…You could portray that you're the greatest athlete out there, but you're not… that you're doing great, but realistically not” (TM-2a). The athletes also commented on specific smartphone functions they used in the sport context (predominantly music, video, social media, and team sport applications). The athletes were particular about smartphone features used in competition, practise, and dry-land training (i.e., gym). Generally, the athletes described more productive and controlled smartphone usage in the sport context, as compared to other contexts such as in class: “[My phone] is certainly on me the majority of times. The only time it's not is if I'm at practise and at the gym, because I want to focus… You know, I wish I was that focused in class” (TM-2b).

#### Restrictions of Usage

Generally, no team-wide, formal regulations existed to dictate the control of smartphone usage in the sport context, and experiences of smartphone rules were not uniform among the athletes. The majority of restrictions that athletes faced were self-imposed, coach-imposed, or based on unwritten rules to maintain respect and live up to social norms: “On our team…about an hour before games, there are no phones. Like, it's not *written* anywhere” (BF-1). One athlete elaborated on the intensity of these social norms among his teammates:

I've gotten mad at people for having their phones out in the room. If you come in at half-time there's no reason to be on your phone checking Facebook… But, we don't have strict rules. Once, I saw a guy check his phone to see a football score after we had just lost—this was 30 s after we got in the room—and somebody just lost it [on him]. (LM-5)

Some athletes described self-imposed smartphone restrictions that helped them with preparation and optimal focus in the sport context.

I basically cut out all social aspects of my phone's use about 20 min before I start warming up, just cut all conversations and say, “I'm going. I'm competing. I'm focused in.” I'll put my headphones in, and then focus my usage solely on my training and competition. (TM-2a)

Interestingly, for some athletes, environmental conditions such as extreme cold and water dictated whether they could use their smartphones at training and competition. Many athletes said they controlled their usage in the sport context simply due to the fact that their phone would be far away in a bag, locker, or change room. In this respect, sport created a natural separation from the smartphone.

The greatest paradox expressed by the athletes pertained to the experience of being separated from their smartphone. Many identified deliberately taking a “break” from their phone as a source of relief. However, this relief was only present if athletes were not expecting or anticipating any important information via their phone. If smartphone separation was forced upon them (e.g., forgetting their phone, phone crashing), this could induce a state of anxiety and/or panic. One athlete explained her dichotomous position:

I think I'm calmer when I know I don't need it. Because I know if I need it, then I'm waiting up checking on it, getting anxious… It's a cross between freedom and anxiety. It's freedom of “I just don't have my phone.” And then anxiety, obviously, if you are expecting something. (RF-2)

### Negative Usage

Negative usage pertained to any experience of smartphone usage deemed debilitative to self-management, optimal functioning, performance, and/or well-being. The three main sub-themes pertaining to negative usage were (a) stress, (b) distraction, and (c) disengagement.

#### Stress

The athletes experienced smartphones as a source stress induced by feelings of obligation, pressure, and FOMO, which all appeared to be intertwined. Stress was induced both by features of the smartphone and obligations associated with the smartphone. FOMO was a major component of stress induced by smartphones. Athletes reported feeling more comfortable when their phones were easily accessible: “I guess it's just a comfort thing, to have it there” (LM-5). Uniquely, athletes reported stress when separated from their phone, for fear of missing out on essential information or updates from their team and coach. One athlete mentioned, “Quite often we'll have to look at our phone to see when our event is, or to double check 500 times to make sure that it still says the same thing, always double checking” (TM-1). The athletes also discussed feeling disappointed about missing out, induced by the social scarifies they have to make as varsity athletes. This feeling was easily exacerbated by their smartphones, which allowed non-athlete peers to put pressure on the athletes to disregard their sport commitments for social events. Athletes commented that their smartphones allowed pressure from the outside world to be brought into various performance situations (e.g., messages from professors during practise), and conversely, pressure from the sporting world intruding in personal situations (e.g., messages from coaches during down time). As one athlete explained:

I find I can't handle the social media at competitions, when I'm already stressed out. I just find texting people and messages to be way too stressful for me. None of my friends who are not at the track care about how my events are going… To me, that's like the outside world at that point, and I don't really want to deal with that. I just want to deal with what's happening at the competition. (TM-1)

A major catalyst of stress was obligation—a pervasive and often overwhelming negative feeling of urgent responsibility. Participants revealed that smartphone usage, and even the mere presence of a smartphone, fostered a feeling of obligation to be accessible at all times, to provide immediate responses, and to provide continuous updates, including on performance outcomes.

I find it stressful sometimes, on Messenger and in a [text] conversation, if I don't want to reply, but have already seen it, it's like: *Oh my god! It says I've seen it, I HAVE to reply now*. Then, you get stressed out. (TF-1)

Participants noted that while this feeling of obligation is likely not unique to athletes, it is intensified for varsity athletes, due to the demands of their coaches in particular. The athletes felt that communication from their coaches was something they could not ignore or save for later. Thus, the athletes agreed that the smartphone communication habits of their coaches would directly implicate their own smartphone habits and sense of obligation to stay connected. One athlete explained, “[As an athlete], I think the requirements of how often you should check your phone are higher… more serious. You don't have a choice if your coach is emailing you; he's not going to wait” (BF-1).

Moreover, several athletes felt that a unique aspect of social media in their lives was the pressure to properly represent their institution. As one athlete explained:

Every team has started an Insta[gram] or Twitter. I think everybody's trying to push that on us…and that differs from the regular student body. We don't even have a choice, really. We're just already implicated… Public space is a public space. So we're implicated whether we want it or not. (RF-2)

Obligation to the university made a number of athletes feel that they had to be active on social media, even if they preferred not to be. The athletes also discussed their obligation to be ever-aware of their social media presence, modelling respectable behaviour. Notably, some athletes felt their university's representation of them on social media was a direct reflection of the institution's support (or lack of support) for their sport. Some athletes described how the university's social media made them feel disregarded and overlooked by the institution. One athlete described being “bumped” out of the way by more popular sports:

We got double banners, beat [our rivals]… We got one post [on social media], which was then bumped 5 min later by a men's game [happening] in 3 weeks… We just did something huge; we creamed them. And we get bumped. (XF-3)

#### Distraction

Participants also discussed their smartphones as a source of distraction that could consume their attention and lead them to engage in absent-minded, non-task-specific thinking or behaviour. They explained that distraction could be related to features of the phone, the mere presence of the phone, and even thoughts about the phone. Athletes' experiences of distraction led to time-wasting, usage regret, and idleness.

I find it gets so easy to just kick back and be like, “Oh, Instagram. Cool picture of windsurfing. Cool picture of kayaking. Cool thing fencing. Cool thing skiing,” and then 2 h go by and I'm like, “I haven't written any of my thesis and it's due tomorrow!” That sucks. (FM-3)

Many athletes also reported that their smartphones distracted them from their ideal sleep routine. It was before bed that athletes felt the least control over their phone usage. One athlete gave an example, “Last night, I was in bed at 10:30 pm and a friend texted me, and it turned into a deep conversation. Two hours later I'm like, ‘Wow, that smartphone just robbed me of 2 h of very precious sleep”' (TM-1). The athletes noted several similar experiences of becoming “trapped” by the constant influx of content on their phones: “Before I go to bed, usually I go on social media. And I think at that point [my smartphone] kind of controls me, because I'm so tired, but I'm still scrolling… even though I really want to go to bed” (SwW-4).

In the sport context, some athletes repeatedly checked phones during short breaks at training instead of staying focused on the task at hand. They explained that during these short breaks, they would become distracted by their phone notifications (both real and anticipated), and would focus on negotiating to the urgency and importance of checking and responding to them. For example, “During that break, you know you'll go get water. So, if I know someone is needing an answer, I'll go run and check it [my smartphone]” (FM-3). Another commented, “It really depends what the message is about, or if I was messaging someone previously, I would message them back. Sometimes, I realise, ‘Will this help me? Or not?”' (XF-1). Moreover, several athletes allowed themselves to be distracted by re-negotiating their self-imposed usage restrictions at training or competition if they were expecting an important e-mail or message, or continuing an important conversation from outside of the sport context.

Once [we] get to the venue and we're in our suits and everything, I usually just put my phone in my bag… Unless I'm waiting for an email back or waiting for a text back or something… I'm pretty bad for that; then I'll usually check my phone. (SwF-4)

#### Disengagement

The athletes also discussed smartphones as a source of disengagement, whereby they were preoccupied with the smartphone to the extent that they were no longer fully participating in the task at hand: “So many times, I'll check [my phone] 1 s and [then] look up, and it's like, ‘Whoa! It's been 5min and I didn't hear anything they said” (XF-4). Athletes admitted to experiencing disengagement themselves, and being impacted by the disengagement of those around them. Specifically, athletes divulged several instances in which parents, coaches, and teammates were disengaged from competition—not cheering or watching an event—because they were preoccupied with their smartphone screen.

It is really hard when you are having a shitty meet or something, and you come back from a race, and you see a bunch of people on their phone. Like, they didn't even watch you race… That can be really frustrating. When other people are zoning out and doing their own thing, it's really hard. (SwF-2)

Some athletes proposed that the disengagement they were noticing was influenced by smartphone temptation, along with a lack of self-awareness and self-control. A few athletes speculated that disengagement at training or competition was not beneficial to performance “I think a lot of people who are on their social media during a swim meet aren't really into the meet, and then they don't perform as well” (SwF-4). One athlete described a perceived link between disengagement and a lack of intensity, which he attributed to excessive smartphone and social media usage:

It's almost like there's no intensity with them [rookies] when they're practising. Like if it's not funny or comical, or doesn't send them a notification that something's going to happen, then they're not concerned about it at all… They're just not there ever, ever. (LM-5)

These experiences of disengagement led some athletes to perceive isolation at competition, as well as low team cohesion and support. Two athletes discussed this experience post-performance, “You come up to someone and they're like, “Oh how did it go?”… *Well, why weren't you watching? You were literally sitting right here!”* (SwF-2). “They devalue what you just did. *Why would you ask that?. Why didn't you just take the time to pay attention if you really care?*” (RF-2). There was a consensus among participants that many people in the sport arena, including athletes and spectators, were more concerned with posting than experiencing the event at hand. Some of the athletes' sentiments included, “Everything now is about showing off to people that you've done shit instead of doing it” (SwM-4), and, “They'll only want to go there so they can get a good picture” (RF-3).

### Positive Usage

Positive usage pertained to any experience of smartphone usage deemed facilitative to self-management, optimal functioning, performance, and/or well-being. The two main sub-themes identified were (a) self-regulation and (b) social connectedness.

#### Self-Regulation

For all athletes, the smartphone functioned as an accessible, efficient multitool that allowed them to self-regulate (i.e., plan, self-monitor, perform tasks, and reflect) in a variety of domains. As one athlete described, “I think [the smartphone] can be very, very useful because it's basically like a super swiss army knife in your pocket” (SwM-3). The majority of participants reported that their phones were an essential tool, and described using the smartphone to successfully manage various aspects of their learning, performance, and day-to-day functioning:

One of the biggest things about being a student athlete is being organised… and [the smartphone] is just a great tool, because it takes less time. And we're already so pressed for time. we all see how it helps, and how it enhances our productivity. (RF-3)

Importantly, smartphones allowed athletes to do this immediately, in any setting, and often remotely, so they felt more in control of their learning and workload when dealing with multiple demands. As one athlete explained:

I used to be a teacher's assistant, and I would get emails [about] my thesis and other projects. After my shower, I would sit down for 20 min in the locker room and just answer every single one…. The smartphone was really, really useful for that, so I could just get through it. (SwM-3)

Participants widely reported the use of smartphones for self-regulation in sport, particularly during independent training and preparation: “I use [my smartphone] every day. I schedule everything. I use it for practise, I log my weights, and my distances in my approaches, and stuff like that” (TM-2b). For example, they talked about using notes and music during sport preparation to increase focus, motivation, and mental readiness. One athlete noted, “For mental preparation, I think all of us use [smartphones] for music, to block sounds from around us… I use music to prepare myself to get focused and list the things I need to focus on while I race” (SwM-4). Another athlete explained:

In my warmup, I heavily use my phone. I'll always have music. I have in my notes who we're playing and people to look out for, their tendencies and points. Then, if it's a bus ride, I'm usually watching a game film of a previous game they played, and looking at their stats. I want to know which hand they're going to shoot with, and who's going to shoot on the power plays. The phone's huge for that, and so accessible. (LM-5)

Some athletes reported using social media to facilitate motivation and goal setting, while video and photo features were used to facilitate self-reflection and evaluation. One athlete explained, “In the summer, I use my phone all the time. I'll video myself doing a movement and then I'll watch it on my phone, see how it went, put it in slow-motion. And then keep practising, doing it again.” (LM-5).

#### Social Connectedness

Although participants reported that constant connexion with others could be a driving source of stress, they also noted the importance of their smartphone for providing meaningful connexion with others and fostering a sense of community. The athletes distinguished the importance of genuine vs. disingenuous connexion, and reported that helpful and positive connexion came from those they perceived to be genuinely interested in their well-being, development, and performance.

Parents were reported to be some of the most important, genuine connexions for the athletes, particularly for those who lived away from home. In several cases, being able to remotely connect with a parent was essential to the athlete's preparation for an event, or for a debrief following it. As one athlete explained, “Sometimes I phone my mum. Sometimes quite a bit, depending on the competition and how stressed out I am. I appreciate phoning my mum or messaging her; it can just bring me back down to fairly steady” (XF-1). Another athlete commented, “Staying in contact is huge… Especially away games, when I walk out of the room, I'll take my phone out and usually I'll call my parents before getting on the bus” (LM-5). Furthermore, one athlete explained the positive way his social media fostered mass, remote communication and support from his family: “It's fun for my family to see. They look at the photos and they're like, ‘Oh, cool! You did this!” (FM-3). Many athletes reported the necessity of their smartphones in fostering this connexion to feel adequately supported in their sport pursuits.

The majority of athletes explained that they used smartphones to foster ongoing team communication: “Communication with the rest of the team, and the coaching staff, and everything—[the smartphone] is vital, and I say that truly understanding the meaning of the word *vital* tool for that” (RF-2). They also used their smartphones to enhance cohesion, fun, and a sense of community among their team members, as well as among other varsity athletes, and athletes from the same sport across the nation. They described that this connexion could occur through group chats, team apps, and social media posts.

Although the athletes reported mixed feelings related to social media presentations, they articulated an understanding of how social media posts could foster a sense of connexion and community for athletes who don't play. One athlete commented on how social media allowed rookies and benched players to feel engaged and proud to be part of the team, even if they did not play: “[Rookies] might have played; maybe they didn't. But, they were dressed for the game, and they'll take a picture and post it after and say, “So happy to be a [name of team]!” It's good! It's good that you want to show that off, and you're proud, and you're still happy, even though you didn't play… Even if you didn't get picked, it doesn't mean you didn't help us get here” (RF-3).

A few athletes explained how positive presentations on social media could serve as a tool for athletes to derive positive gains from a difficult outcome: “But, we do that [positive social media posts] because we're trying to be positive. Our coach always says, ‘It was a good meet! It was hard!' … People are just trying to be positive and hype it up a bit more” (SF-3). One athlete commented her fellow athletes will often use social media platforms to candidly share about losses and difficult performance outcomes, in order to seek support and connexion from their sport community.

Because it's such a small sport, people are constantly saying, “Oh I didn't perform, but this is why….” So often you'll see photos of, “Rough weekend of racing; didn't have my legs”… You know people are saying it to make themselves feel better, because [it] was a great race but you didn't like your results. (XF-3)

## Discussion

The aim of this study was to explore varsity athletes' positive and negative smartphone experiences, with the intent of providing insight into how they are using their smartphones and how their usage is impacting their experience of being a varsity athlete. Results show that smartphones were highly used by the sample of varsity athletes, with 81% of them self-identifying as being moderate or heavy users, relying on their device throughout the day. Moreover, smartphones were largely used for social media, communication, and organisation. This is congruent with characteristics of the general population, and particularly the iGen (Bentley et al., 2015; Smith, 2015). Furthermore, varsity athletes' experiences in sport were perceived to be influenced by their smartphone usage. Positive and negative implications of usage were shared by the athletes and will be addressed in the following three sections: (a) negative and positive smartphone usage, (b) continuum of smartphone usage, and (c) applied considerations.

### Negative Usage

Athletes perceived their smartphones to be a source of stress when their phone was both available and unavailable to them. This is consistent with the literature that points to mobile phones causing increased stress, pressure, and overload particularly through constant access to information, connexion, and demands (Thomée et al., 2010). Participants reported feelings of anxiety when separated from their smartphones, and several studies have shown that stress and anxiety can result from being separated from smartphones after only a short period of time, particularly within the iGen, and among heavy smartphone users (e.g., Thomée et al., 2010; Rosen et al., 2013; Cheever et al., 2014; Clayton et al., 2015). This could help explain the athletes' reports of constant checking behaviours at competition and practise. Clayton et al. (2015) have studied psychophysiological outcomes of smartphone separation, and found that when participants were unable to answer their phone while performing another cognitive task, “heart rate and blood pressure increased, as well as feelings of anxiety and unpleasantness” (p. 123). If anxiety, psychological intensity, and physiological arousal are heightened by smartphone separation, it warrants keen attention from the sport community. Smartphone separation anxiety could inhibit optimal performance states by unnecessarily raising athletes' anxiety and arousal, particularly among those who are heavy users and have developed dependence on their phones (Cheever et al., 2014).

Specific feelings of being obligated to communicate with coaches and to positively represent the university online appear to be unique additional stressors for varsity athletes, which can be exacerbated by their constant connexion to smartphones. These findings are in line with literature highlighting varsity athletes' perceived obligation to maintain a curated image online (David et al., 2018; Sanderson, 2018; Park et al., 2020). The athletes in this study experienced unique, sport-specific FOMO based on the stress of missing essential information from coaches or information that could potentially impact their sport performance or success (e.g., starting lines, different race times). Research indicates FOMO can drive increased social media and smartphone usage, thus compounding demands, incurring detriments to self-control, and influencing distracted and multitasking behaviours (Przybylski et al., 2013; Clayton et al., 2015). This added pressure from fear of missing essential sport information, could be influencing varsity athletes to use their phones more than they otherwise would. Additionally, researchers have proposed that habitual checking and fear of missing important information can lead to the development of problematic smartphone usage (Elhai et al., 2017, 2019). This suggests that through added demands and obligations in the varsity sport context, athletes may face unique, additional risk factors for problematic smartphone usage.

Smartphones were also a distraction for participants, leading to absent-minded usage among the athletes, particularly before bed and in “waiting times.” This aligns with literature pointing to smartphones (and accompanying features) as catalysts of internal and external distraction, absent-minded usage, inattention, sleep disruption, and media multitasking, which can be debilitative to performance and well-being (e.g., Thomée et al., 2010; Clayton et al., 2015; Stothart et al., 2015; Carrier et al., 2018; Marty-Dugas et al., 2018). Results also show that in the sport context, some athletes sporadically put energy and attention towards negotiating the importance of smartphone checking and notifications. Stothart et al. (2015) found that just the receipt of a phone notification can be detrimental to attention and performance, likely by influencing task-irrelevant thoughts. Further, Ward et al. (2017) argue that cognitive capacity can be depleted by the presence of a smartphone, even when people control their attention and are not consciously tempted to check their phones. This suggests that the cognitive capacity of athletes could be at risk, even if they are actively choosing not to engage with their phones. This is also salient because smartphone and social media usage prior to competition and training has been found to incur concentration disruption and inhibited decision-making in athletes (Encel et al., 2017; Fortes et al., 2019). It appears that if unmanaged, internal and external smartphone distraction has the potential to be cognitively depleting, and disruptive to athletes' concentration and performance (Encel et al., 2017; Fortes et al., 2019).

Notably, athletes did not report experiences of smartphone distraction within their own sport performances (e.g., when running on the track, swimming in the pool, or playing on the field). This is likely because in active training and competition situations, athletes are naturally, physically separated from their smartphones, a characteristic of the sport setting that was highlighted by participants. Ward et al. (2017) have shown that those with full separation from their smartphone had better cognitive task performance, even compared to those who had their smartphone on their desk or in their bag on “silent”. These findings suggest that distraction may be further influenced by the vicinity or “salience” of the smartphone (Ward et al., 2017). Sport may well provide a unique arena where smartphone distraction could be managed by the natural separation of athletes from their phones. However, in order to fully benefit from this, athletes would need to train and utilise strategies to maintain task-focus and reduce smartphone dependence, including anticipation of notifications or future usage (Clayton et al., 2015; Stothart et al., 2015; Ward et al., 2017).

Results also point to smartphones as a source of disengagement in the sport context. Athletes reported that the disengaged behaviour of friends, family, coaches, and teammates, due to their use of smartphones during training and competitions (i.e., not being present or attentive to the situation at hand and concentrating on one's phone instead), caused detriments to athletes' sense of team cohesion and support. In line with this, research suggests that smartphones are influencing interference and disengagement in social situations (Chotpitayasunondh and Douglas, 2016; McDaniel and Coyne, 2016). Interestingly, research has shown that FOMO and lack of self-control—two characteristics that emerged from our results—may be predictive of smartphone addiction and lead to preoccupation with smartphones instead of attending to the present social situation (Chotpitayasunondh and Douglas, 2016). Some athletes commented that self-control is lacking among their teammates, and a few blamed the disengagement and diminished performance of rookie players on smartphone usage. It seems that the athletes' perceptions may be founded, as studies have uncovered that those who use their phones more exhibit poorer self-control and efficiency, more impulsive decision making, less analytical thinking, and inattention (Abramson et al., 2009; Marty-Dugas et al., 2018). Strengthening self-control, self-confidence and self-determination (to reduce fear), and attentional control may be important strategies to improve athlete engagement.

### Positive Usage

Aside from negative implications of usage, results point to the benefit of using smartphones to build team cohesion, as well as a sense of community and support, particularly through ongoing connexion with peers and family. This is in line with literature that points to young people using a variety of smartphone applications for relationship building, connexion, social interaction, and coordination (Gardner and Davis, 2014; Bentley et al., 2015). Connexion was particularly important to the varsity athletes, who found themselves at a distance from their team and core social supports (e.g., when on the road, not playing due to injury, or being benched as a rookie athlete). This is congruent with Frison and Eggermont's (2015) assertion that social media is an important means of social and emotional support among young people, specifically when the needs of the support seekers are adequately met by the support network. Furthermore, Wright (2012) found that emotional support through Facebook predicted lower perceived stress among college students, suggesting that social media support among athletes could also have a positive buffering effect on their perceptions of daily stressors. This may be particularly relevant for varsity athletes, as Wright (2012) determined that *homophily* (i.e., similar attitudes and backgrounds) among users increased their perceptions of support through social media. The results from our study show that social media may provide a unique space for athletes to debrief, reframe, and seek support following difficult sport events and outcomes. Taken together, the use of smartphones and social media for connexion, community-building, and social support among varsity athletes could foster opportunities for enhanced well-being, and help to mitigate pitfalls of varsity athletics such as increased stress and isolation (Dubuc-Charbonneau and Durand-Bush, 2015).

Another positive outcome of smartphone usage was augmented capacity to self-regulate in the face of multiple demands and contexts (e.g., sport, school, personal life). This is in line with other literature highlighting the multi-faceted use of smartphones among young people (Gardner and Davis, 2014; Bentley et al., 2015; Smith, 2015). The athletes' smartphones supported the optimization of self-regulated learning processes, particularly planning, self-reflection, and self-presentation in the sport setting. This is not surprising given that a number of interventions in health settings have shown the benefits of smartphones as self-regulatory tools for self-monitoring, goal setting, and self-reflection (e.g., Quelly et al., 2015). A great deal of literature has focused on the detriments of smartphone usage, but much less has focused on the potential of smartphones to support self-regulatory learning processes, which are essential components of athlete functioning, development, and performance (Cleary and Zimmerman, 2001; Dubuc-Charbonneau and Durand-Bush, 2015). The varsity athletes in this study found their smartphones to be essential tools to fulfil multiple roles and responsibilities. In this sense, self-regulatory smartphone usage could act as a buffer to the unique demands of being a student-athlete by supporting self-regulation within and outside of the sport context.

### Continuum of Usage

Results of this study suggest that a complicated, paradoxical relationship exists between athletes and their smartphones. While all athletes felt that their smartphones were essential tools, they identified a dichotomous or “torn” feeling about their usage that was heavily dependent on context, purpose, and time of usage. For example, smartphones may have been helpful for self-regulation, but they concurrently interfered with self-control and cognitive capacity. The devices also fostered community building and social support, but equally led to distraction and disengagement, inhibiting team cohesion and perceptions of support. Equally, smartphones may have given student-athletes a sense of control over the multiple demands they faced (e.g., sport, school, personal life), all the while fostering stress, FOMO, and a sense of obligation. While there are both positive and negative implications of smartphone usage for varsity athletes, these implications may exist along a continuum, rather than within succinct, polarised categories. Although the athletes shared common experiences and perspectives of smartphone usage, their personal preferences, habits, and outcomes of usage were nuanced and idiosyncratic across contexts and situations. Similarly, Chan and colleagues (2015) reported a dichotomy in the lived experiences of students' smartphone usage for learning purposes. The authors also introduced the notion of a continuum of “serendipitous and purposive” mobile learning, shaped by time and intent of usage (p. 101). A similar continuum appears to be applicable to this cohort of varsity athletes, pointing to helpful and unhelpful aspects of usage that fluidly coexist. According to student-athletes in this study, context of usage is another important dimension to consider within this continuum of usage. A few other studies examining how social media and smartphone usage impact well-being underscore similar convoluted areas (e.g., Best et al., 2014; Ryan et al., 2017; Elhai et al., 2019; Ellis, 2019). This suggests that the use of smartphones should not categorically be deemed as good or bad. Instead, these devices offer a double-edged value for varsity athletes that is highly dependent on time, purpose, and context of usage.

### Applied Considerations

It is clear that varsity athletes use their smartphones to manage roles and demands across multiple contexts (e.g., sport, school, home), and so, simply focusing on the negative implications of usage does not acknowledge the full range of athletes' interactions with their phones. Building on this, and in line with Durand-Bush and DesClouds's (2018) suggestions for smartphone usage in the sport context, it is recommended that sport psychology practitioners, coaches, and athletes avoid a one-size-fits-all approach to smartphone rules and regulations. Instead, athlete autonomy and accountability for smartphone usage should be promoted through consistent, open dialogue about how smartphones can help and hinder sport performance and experiences. These discussions can inform the creation of individualised guidelines for smartphone usage that carefully consider the context (e.g., training, competition, school, home), purpose (e.g., planning, self-presentation, entertainment), time (e.g., morning of competition, bedtime) of usage, as well as individual goals, needs, preferences, and self-regulation skills. Through such guidelines, the benefits of smartphone technology (e.g., to support self-regulated learning) could be carefully leveraged, and restrictions could be put in place in areas where phones tend to have a negative impact.

Results of this study show that athletes had a high level of self-awareness and were deeply in tune with the various facilitative and debilitative implications of their smartphone usage, as well as their internal dialogue cautioning them about their media habits. However, they appeared to lack self-control in certain situations. In line with research showing young user's acute awareness of usage, and inclination to use their phone in unscheduled times (e.g., between tasks, when bored, and while waiting; Bentley et al., 2015), the athletes in this study reported using their phones when bored, procrastinating, and between tasks. The athletes specified engaging in passive, absent-minded usage during these times, arguably leading to attentional deficits and cognitive depletion. Research is pointing to mindfulness, meta-cognition, and self-regulation as positive coping strategies to mitigate the negative effects of smartphone usage (Carrier et al., 2015; Bauer et al., 2017). Sport psychology practitioners are uniquely positioned to help athletes and coaches develop such skills and gain insight into the effects of passive (absent-minded) vs. active (purposeful) usage. Establishing mindful, purpose-driven smartphone usage plans to optimise performance and well-being appears to be a worthwhile endeavour when working with varsity athletes. Furthermore, given that many varsity sport departments now have policies regarding the use of social media, it would be important to inform them of relevant research findings to support and adapt their policies to optimise both social media and smartphone usage.

### Strengths, Limitations, and Future Research

There is a paucity of studies on the impact of athletes' smartphone usage. While this study is the first to explore smartphone usage within the context of Canadian varsity sport, it is time-limited by rapidly evolving technology and literature. The sample is limited both in terms of the number of participants and the contexts from which athletes were recruited. It is possible that some athletes' perspectives (e.g., across different sports, genders, and years of eligibility) were not represented in the focus groups. Consequently, broad generalisations should not be made. Nonetheless, the results of this study give insight into the complex and nuanced experiences that varsity athletes may have with their smartphones. It is clear that there are both benefits and drawbacks from using these devices in and around the sport context, leading athletes to perceive a complex relationship with their smartphones. This manuscript constitutes a first step in research on the impact of smartphone usage in athletes' lives, and can inform a breadth of future studies in this area. This study also provides a foundation for the development of evidence-based guidelines for smartphone usage in sport, which consider the full range of helpful and unhelpful uses of this technology.

Findings can inform future research in a number of ways. For example, studies should aim to investigate the longitudinal impact of smartphone usage on athletes, considering variables such as time, purpose, and context of usage. Researchers should also examine specific characteristics (e.g., frequency and types of features being used on the smartphone) and outcomes of usage, including sport performance and well-being. Moreover, social media was a prevalent feature of the athletes' smartphone experiences, used for sport-specific purposes such as self-monitoring, reflection, comparisons, and self-presentation. As such, research should continue to explore the role of social media in athletes lives, including the role of social media within the self-regulatory processes of athletes. Given the dichotomous findings regarding self-regulation and self-control, scholars should look at the interplay between smartphone usage and these variables. Finally, studies should focus on the potential of smartphones to foster self-regulatory learning in the sport context, to better understand whether and how purpose-driven usage might enhance sport learning, performance, and overall mental health.

## Conclusion

Smartphones are powerful, omnipresent devices capable of generating both negative and positive experiences in the sport context. Negative implications of usage in this study included stress, distraction, and disengagement, while positive influences involved self-regulation and social connectedness. Smartphones concurrently offer challenges and opportunities for varsity athletes, underscoring the importance of effective self-regulation and self-control to leverage the benefits and mitigate the risks of these devices. Athletes' relationship with their smartphone is complicated and nuanced, suggesting that usage is best viewed on a continuum, rather than through two polarised ends. Time, purpose, and context of usage are important variables to consider when exploring the impact of smartphones on varsity athletes.

## Data Availability Statement

The datasets generated for this article are not readily available because approval from the Research Ethics Board is required in order to release any data from this project. Requests to access the datasets should be directed to Poppy DesClouds, poppy.desclouds@uottawa.ca.

## Ethics Statement

The studies involving human participants were reviewed and approved by The Health Sciences and Science Research Ethics Board, Office of Research Ethics and Integrity, University of Ottawa. The patients/participants provided their written informed consent to participate in this study.

## Author Contributions

PD conceived and carried out the research project in consultation with ND-B, and wrote the manuscript. ND-B supervised the project, provided critical feedback, and edited the manuscript. All authors contributed to the article and approved the submitted version.

## Conflict of Interest

The authors declare that the research was conducted in the absence of any commercial or financial relationships that could be construed as a potential conflict of interest.
